# Reframing research impact

**DOI:** 10.1016/j.patter.2022.100508

**Published:** 2022-05-13

**Authors:** Amy P.K. Nelson

**Affiliations:** 1 High Dimensional Neurology Group, UCL Queen Square Institute of Neurology, University College London, Russell Square House, Bloomsbury, London WC1B 5EH, UK

## Abstract

Amy Nelson, Senior Research Associate at University College London, and her team proposed a suite of deep learning models for scientific research evaluation that goes beyond citation-based features in impact analysis of biomedical research. In this People of Data, she talks about the future of medicine and patient care from the perspective of data science.

## Main text

### What would you like to share about your background (personal and/or professional)?

Thanks for the opportunity to feature our work. I’m a medical doctor and Senior Research Associate working in the High Dimensional Neurology Group at UCL Queen Square Institute of Neurology, led by Prof Parashkev Nachev. The study published in this issue^1^ is part of my role to develop models of research impact. In parallel, I am involved in the application of complex modeling to operational problems in hospital healthcare, a surprisingly neglected area where contemporary machine learning can rapidly add value. For example, we recently showed that we can predict non-attendance with fidelity high enough to save ∼£3 per appointment, a substantial number considering a large hospital will typically see hundreds of thousands of scheduled events per year.^2^ The need for systems of this kind was very apparent to me during my junior medical jobs, and catalyzed my interest in applying machine learning to clinical problems. In terms of training, I completed my medical degree and Pharmacology Honors at University of Edinburgh, where I had the fortune to undertake projects at some excellent labs including with Prof. Herbert Chase at Columbia University’s Biomedical Informatics Department, Dr. Suvankar Pal and Prof Rustam Al-Shahi Salman at the University of Edinburgh, and Prof. Clemens Löwik at Leiden University Medical Center.

### How did this project you wrote about come to be?

As the Research Impact Fellow for the NIHR UCLH Biomedical Research Center, I was tasked with improving how we measured our own translational (real-world) impact. Our BRC leadership team were forward-thinking about the issues involved and commissioned us to take a broad, theoretical approach. In setting our direction, we were invaluably aided by discussions at the Association of Medical Research Charities Impact Coffee Club meetings, where it was clear that many major research bodies were struggling to define translation with enough scope that it could be systematically measured without losing the depth needed to capture the nuances of medical research. My co-authors and I proposed “ground truths” that signify a link between research and a real-world effect, which became concretized to a paper’s inclusion in either a patent, or guideline or policy document reference list—chosen to reward basic science and clinical research respectively. The first “ground truth” was available in Microsoft Academic Graph, which had been made publicly available just two years previously; the second was made possible through a fruitful collaboration with Wellcome Data Labs, who had been collecting these data toward aligned aims. With these pieces in place, we were able to start reframing the measurement of research impact as a predictive task to which we could recruit a host of deep learning methods. It was an exciting moment!

### Was there a particular result that surprised you, or did you have a eureka moment? How did you react?

One particularly challenging aspect of this project was working out how to embed the content of titles and abstracts in a way that utilized contextual meaning within the text, rather than just registering the use of specific words. Most previous approaches at the full-domain scale used exactly that: word embeddings, and few solutions existed that satisfactorily applied rich learnt language embeddings at the paragraph level. To solve it, we extracted sentence embeddings using a BERT model pretrained on biomedical papers (BioBERT) and concatenated each paper’s title—and the first 20 sentences of its abstract—as input to a CNN with initial stride length equaling the size of each sentence embedding. This provided a much richer representation than simply averaging across sentences, and led to one of the findings that I was most excited about: that translational research impact, measured by patent, guideline, or policy inclusion, can be predicted based on content alone.^1^ To me, that got to the core objective of bibliometrics—we weren’t just picking up signal biased by previous citations, or institutional prestige; there was something intrinsic to the language of science denoting a paper’s translation potential. My first reaction was caution, which thawed after seeing the beautiful (to my eyes) unsupervised separation of classes on deep autoencoder clustering!

### Why did you decide to publish in *Patterns*?

*Patterns* has wide reach across data science expertise—both fundamental and applied—facilitating rigorous criticism of the work it publishes. Our own work received many insightful comments from a multiplicity of perspectives, returned within 3 weeks! We also liked the forward-thinking perspective of the journal, manifested in features like supplying data science maturity levels to highlight the technology readiness of published work. Finally, given the framing of our work within such a broad research domain, we wanted our audience to view it from as broad a background as possible.

We came for these reasons, but we would publish again with *Patterns* because of the rapid turnaround times, professional staff, engaged and enthusiastic editors, and all-round excellent experience!

### What motivated you to become a (data) researcher? Is there anyone/anything that helped guide you on your path?

Working as a doctor, I was struck by the sheer volume of information collected in the course of patient care, and at the same time, it was very clear that the hospital system was struggling to cope with variations in demand and staffing, and that lessons learnt in some centers were not easily shared with others. While it is very common for individual clinicians to conduct audits and improvement projects, there was no way to learn patterns across the entire system. More widely, the whole approach to answering questions in medicine seemed not to be systematically evaluated or actioned, still mostly driven by the experience of individual or groups of individual clinicians. And yet the complexity of medicine is such that it is likely subject to patterns beyond the intuition gained in a single lifetime of experience. I love mathematics and needed little incentive to study machine learning, which I hoped might allow me to get involved with solving some of these problems. Thankfully, I was helped by many wonderful mentors in the process, including Pearse Keane, Maxine Mackintosh, Herbert Chase, my colleagues at the HDN group, and my PhD supervisors Geraint Rees, Lisa Cipolotti, and Parashkev Nachev. I am also indebted to the amazing people who dedicate time to online math, programming and data science resources to guide people like me on this path.

### What is the definition of data science in your opinion?

I suppose the term is so broadly applied that its definition is open to opinion. For me, it marks the point of departure from hypothesis testing approaches that make advance assumptions about the relationship of independent variables to a dependent variable: e.g., instead of testing whether red wine is significantly associated with cancer, data science approaches would try to learn across a wide array of variables which combinations are important in predicting cancer. This perspective acknowledges that the space of possible hypotheses is often far too wide to be intuitively surveyable, so the rejection of any null is rarely grounds for accepting the alternative. Licensing greater model flexibility further allows us to get closer to the individual—a matter of great importance in medicine, whose primary focus is the patient.

### Which of the current trends in data science seem most interesting to you?

From a clinical perspective, perhaps the most exciting direction of data science is toward causal modeling. Although causal estimation from observational data has been established for some time—the combination of instrumental variables with potential outcomes was awarded the 2021 Nobel Prize in Economics—the use of increasing volumes of data and high capacity models might offer greater insight to complex causal questions in medicine, of the form “what effect does X drug have on Y condition?”. The gold standard in medicine for these questions is the prospective double-blind randomized controlled trial. However, these studies are expensive; are sometimes not possible due to ethical constraints, e.g. in the absence of equipoise; and do not capture heterogeneous treatment effects since they measure average effects across the population. Where evidence gaps exist and there are legitimate contraventions to prospective trials, we may be able to make more informed decisions about interventions using models developed for estimating causal effects. I hope there will be more consideration given to their application and prospective validation in medicine moving forward.

### What is the role of data science in your domain/field? What advancements do you expect in data science in this field over the next 2–3 years?

Within healthcare, the medical imaging field has made the most substantial data science advances, in part due to the large quantity of readily analyzable data collected in the course of clinical care, in part due to the maturity of existing computational methods in the field, and in part due to greater coherence of imaging data to more general experimental paradigms in data science. We’ll continue to see more prospective testing of medical imaging models in clinical environments over the next few years and be able to further quantify the impact of these approaches on patient care.

There is much work to be done in healthcare more broadly: medical imaging accounts for only a small fraction of the information used to diagnose, prognosticate and treat patients – much is spoken, written or observed physically. The intellectual heft of medicine is less classification or regression, more anomaly detection, causal effect estimation, and paradigms yet unexplored. Over the next few years, I hope we’ll also see further progress in natural language processing for unstructured medical text, given breakthroughs in language representation in the wider data science community, and greater exploration of multimodal inputs for clinical models. Useful progress will require more shared learning between clinical and data science experts, creating models that are fully informed by clinical problems, rather than just transferred from more general paradigms.

### What is the fun part of being a data scientist?

It is really satisfying to distil insights from what starts as an overwhelming scale of data: finding meaning in patterns is rewarding to most humans, and data science, like all science, is a structured extension of that. I think it’s also necessary, given the amount of time we spend doing it, to get a buzz from cleaning and organizing data thoroughly. There’s no shortcut to opening the raw datasets to examine inconsistencies, hypothesize reasons for missing values, check for variables that might leak non-informative signal, and get a sense of the density of information, in addition to the more systematic preprocessing steps. This is one of the areas that having dual data science and domain expertise can become hugely valuable: beyond just making problem-informed architectural decisions—knowing the best imputation strategy, or how to compress information effectively, or having priors for how much information we expect to gain from certain combinations of variables, can make a big difference to performance down the line.

### What’s next for the project? What’s next for you?

I’d love to share our work with other organizations who seek to measure the translational potential of their own research or themes, and to spark discussions on the utility of deep metrics in place of simpler decision tools like paper citations when we have real-world impact in mind. At the moment, such impact is typically acknowledged in the form of anecdotal evidence (case studies), whereas we have demonstrated the ability to predict wider impact that researchers themselves may be unaware of. In terms of development, we are keen to test the prospective performance of our models, to explore their equitability, and to build a robust tool for any researcher to measure the impact of their work.

As for me, I’ll be continuing to work in the overlap between data science and clinical medicine, on the lookout for ways to be useful in either.
